# Supplementary material for: Saccharomyces boulardii (CNCM I-745) improves intestinal damage in sepsis by remodeling intestinal flora structure
Source: Front Cell Infect Microbiol. 2025 Jul 31;15:1602792. doi: 10.3389/fcimb.2025.1602792 (PMC12350329; doi:10.3389/fcimb.2025.1602792)
Supplement: Supplementary file 1 [file DataSheet1.zip › summary/summary/8_Advanced_analyse/2_PICRUSt2_result/KEGG/Group.BW0_vs_AW0/PICRUSt2_KEGG_level3_Group.BW0_vs_AW0_diff.pdf]

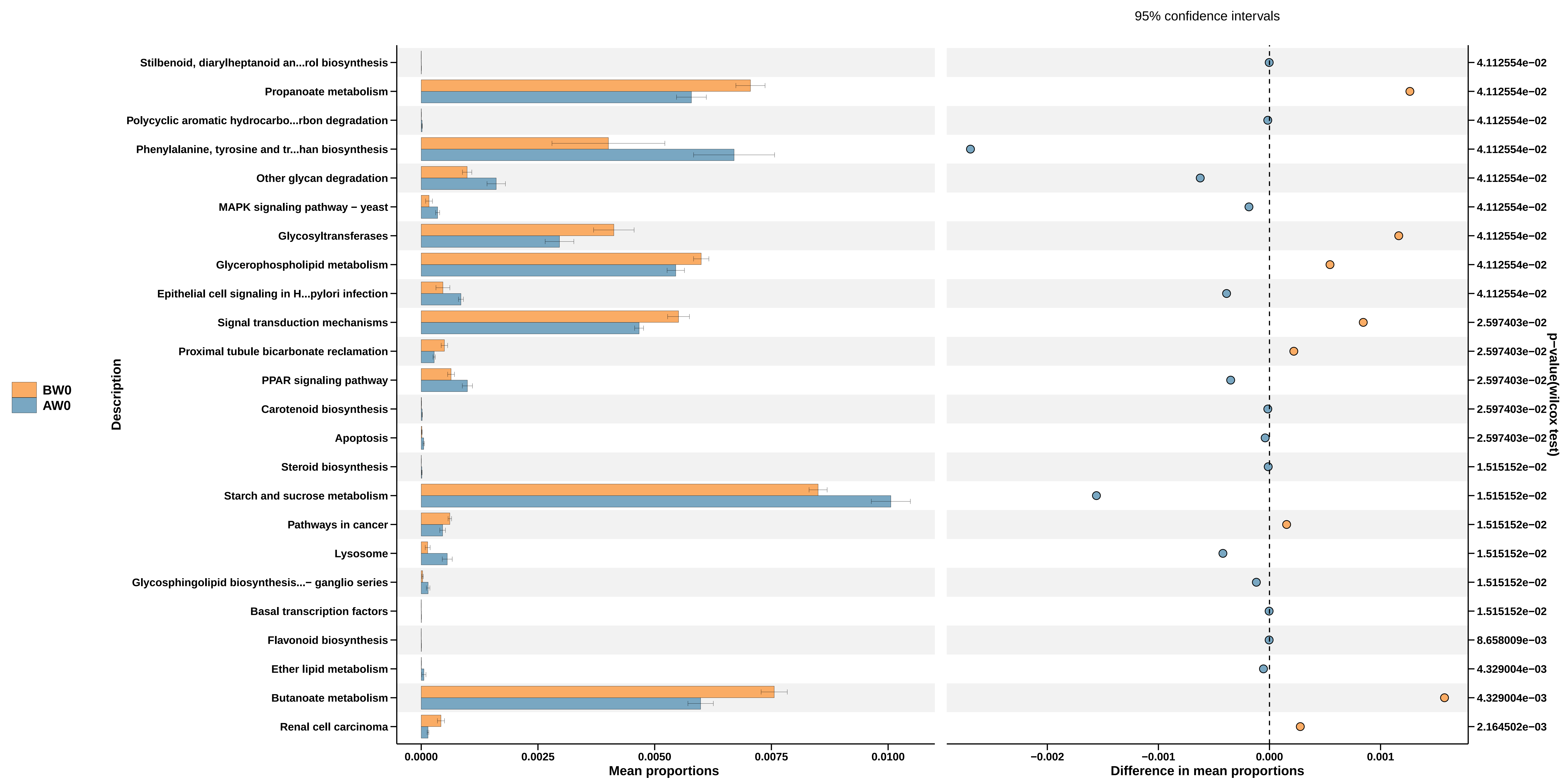
95% confidence intervals
BW0
AW0
Description
Stilbenoid, diarylheptanoid an...rol biosynthesis
Propanoate metabolism
Polycyclic aromatic hydrocarbo...rbon degradation
Phenylalanine, tyrosine and tr...han biosynthesis
Other glycan degradation
MAPK signaling pathway − yeast
Glycosyltransferases
Glycerophospholipid metabolism
Epithelial cell signaling in H...pylori infection
Signal transduction mechanisms
Proximal tubule bicarbonate reclamation
PPAR signaling pathway
Carotenoid biosynthesis
Apoptosis
Steroid biosynthesis
Starch and sucrose metabolism
Pathways in cancer
Lysosome
Glycosphingolipid biosynthesis...− ganglio series
Basal transcription factors
Flavonoid biosynthesis
Ether lipid metabolism
Butanoate metabolism
Renal cell carcinoma
0.0000
0.0025
0.0050
0.0075
0.0100
Mean proportions
−0.002
−0.001
0.000
0.001
Difference in mean proportions
p−value(wilcox test)
4.112554e−02
4.112554e−02
4.112554e−02
4.112554e−02
4.112554e−02
4.112554e−02
4.112554e−02
4.112554e−02
4.112554e−02
2.597403e−02
2.597403e−02
2.597403e−02
2.597403e−02
2.597403e−02
1.515152e−02
1.515152e−02
1.515152e−02
1.515152e−02
1.515152e−02
1.515152e−02
8.658009e−03
4.329004e−03
4.329004e−03
2.164502e−03
